# Endothelial Glycocalyx in Aging and Age-related Diseases

**DOI:** 10.14336/AD.2023.0131

**Published:** 2023-10-01

**Authors:** Lina Sun, Lingyan Wang, Kaisy Xinhong Ye, Shoushi Wang, Rui Zhang, Zhaodong Juan, Lei Feng, Su Min

**Affiliations:** ^1^School of Anesthesiology, Weifang Medical University, Weifang, China.; ^2^Department of Anesthesiology, the First Affiliated Hospital of Chongqing Medical University, Chongqing, China.; ^3^Yong Loo Lin School of Medicine, National University of Singapore, Singapore, Singapore.

**Keywords:** endothelial glycocalyx, aging, age-related diseases, healthy, older adults

## Abstract

The worldwide population is aging exponentially, creating burdens to patients, their families and society. Increasing age is associated with higher risk of a wide range of chronic diseases, and aging of the vascular system is closely linked to the development of many age-related diseases. Endothelial glycocalyx is a layer of proteoglycan polymers on the surface of the inner lumen of blood vessels. It plays an important role in maintaining vascular homeostasis and protecting various organ functions. Endothelial glycocalyx loss happens through the aging process and repairing the endothelial glycocalyx may alleviate the symptoms of age-related diseases. Given the important role of the glycocalyx and its regenerative properties, it is posited that the endothelial glycocalyx may be a potential therapeutic target for aging and age-related diseases and repairing endothelial glycocalyx could play a role in the promotion of healthy aging and longevity. Here, we review the composition, function, shedding, and manifestation of the endothelial glycocalyx in aging and age-related diseases, as well as regeneration of endothelial glycocalyx.

The combination of greater life expectancy and reduced fertility rates has led to an ‘aging population’ where the number and proportion of older adults have increased rapidly. The World Population Prospects 2022 (https://population.un.org/wpp/) issued by the United Nations showed that currently, there are more than 1 billion adults aged 60 years and above globally. Moreover, by 2050 the population of adults over the age of 60 would grow to more than 2 billion in number, with 450 million people over the age of 80. According to the United Nations' World Population Ageing 2020 Highlights (www.un.org/development/desa/pd/content/ageing-1), it is expected that by 2050, 1 in 6 people in the world will be over the age of 65. While the increasing longevity of the human population is something worth celebrating, it is concerned that health does not extend with life expectancy. Older adults often suffer from frailty, deterioration of organ function, poor compensatory capacity, and degenerative diseases. Aging is a multifaceted phenomenon that is observable at molecular, cellular, and physiological levels. Identifying and understanding potential biological targets of healthy aging may allow us to develop preventive strategies to help delay the onset of age-related diseases, hence extending healthspan and improving quality of life.

In recent years, it has been widely recognized that the surface of a healthy endovascular lumen is covered with gelatinous villous material. This layer of proteoglycan polymer is called endothelial glycocalyx (EG). EG serves as a critical signaling platform to integrate chemical and haemodynamic signals [1]. EG plays an important role in regulating vascular permeability, and is involved in mechanical signal transduction, inflammation, oxidative stress, and the inhibition of coagulation and cell adhesion. The integrity of EG is crucial for the maintenance of normal physiological function of the organs. A thinner EG has been commonly associated with age-related chronic diseases [2-4]. This article reviews the composition, function, shedding, and manifestations of EG in aging and age-related diseases, and the potential role of EG regeneration in promoting healthy aging.

## Composition and Function of EG

EG is mainly composed of glycosaminoglycans (GAGs), proteoglycans (PGs), glycoproteins (GPs) and plasma proteins. It is connected to endothelial cells through a cytoskeleton composed of PGs and GPs [5]. The thickness and structure of EG may vary across different species and organs [6]. Determining the thickness of EG is difficult as it can be easily disturbed during vessel handling and preparation protocols [7], leading to inaccurate measurements. However, in vivo immunolabeling and laser scanning confocal microscopy may be effective methods for measuring EG [8].

GAGs are the largest constituent of EG. The various groups of GAGs include heparan sulfate (HS), chondroitin sulfate (CS), keratan sulfate (KS), Dermatan sulfate (DS) and hyaluronic acid (HA) [7]. Among them, HS is the most abundant form accounting for 50-90% of the total GAGs [9]. Due to the highly sulphated nature of these components, EG has a net negative charge. On the other hand, PGs are composed of Syndecans and Glypicans, which are the membrane scaffold of GAGs [10]. Syndecans are transmembrane receptors with four members: syndecan-1, -2, -3 and -4, whereas Glypicans include six members: glypican-1, -2, -3, -4, -5 and -6.

Through covalent bonds, the core proteins of PGs can bind to a variety of GAG chains, primarily with HS and CS, and with DS and KS to a lesser extent [11]. These structures play an important role in signal transduction. HA is an uncharged non-sulfate that binds to the osteopontin receptor CD44 [12]. It is worth noting that aging and neurodegeneration alter sulfation in a fashion that is unpredictable. Therefore, there is a growing interest in finding out exactly how GAGs sulfation affects microvascular physiology in aging. EG can bind with plasmonic proteins and ions with a positive charge as it is negatively charged, and this affects the interaction between platelets and red blood cells [13]. These interactions play an important role in regulating the balance of fluid inside and outside blood vessels and is closely related to vascular permeability [5]. EG can repel negatively charged molecules and form an electrostatic barrier to plasma constituents, like a giant molecular sieve [14]. Studies have shown that the negative charge of EG can be neutralized by myeloperoxidase, and this neutralization affect vascular endothelial function and increase vascular permeability [15]. Macromolecules with molecular weight greater than 70KDa have difficulty penetrating the EG [14]. EG is also a mechanical sensor of blood shear force [16], playing a major role in mechanosensing and transducing in microvessel [17]. It transmits shear forces to endothelial cells via transmembrane domains and converts mechanical stimuli into intracellular signals that controls endothelial function. EG regulates a variety of important physiological functions such as the activation of endothelial nitric oxide synthase (eNOS) and mediates the release of nitric oxide (NO) [18, 19], where glypican-1 and HS are the main receptors that induce the regular release of NO [20]. EG can also combine with enzymes that scavenge oxygen radicals, such as extracellular superoxide dismutase (SOD), to maintain the bioavailability of NO and reduce oxidative stress [21].

GPs are the backbone proteins of EG, consisting of small branches of glucose side chains on the surface of endothelial cells, mostly ending with sialic acid (SA) [9]. GPs include members from the selectin family, integrin family, and immunoglobulin superfamily, each with a different role in cell adhesion. The selectin family is involved in the adhesion of leukocytes and endothelial cells [22], whereas the integrin family mediates the adhesion of endothelial cells to platelets. The immunoglobulin superfamily on the other hand is a ligand of integrins that mediate the return of leukocytes to the endodermis. Members of the superfamily mainly consist of intercellular adhesion molecules (ICAM), vascular cell adhesion molecule-1 (VCAM-1) and platelet endothelial cell adhesion molecule-1 (PECAM-1). If the integrity of EG is damaged, cell adhesion molecules will be exposed, and leukocytes and platelets will adhere to endothelial cells, and lead to platelet aggregation, oedema and loss of vascular reactivity [23].

## Shedding of EG

Under normal conditions, EG is shed under the influence of biological factors and then regenerated by endothelial cell synthesis, maintaining its integrity through homeostasis. The enzyme that degrades EG includes matrix metalloproteinases (MMPs), A disintegrin and metalloproteinase (ADAM), heparanase (HPSE) and hyaluronidase (HAase) etc. MMPs are zinc-containing endopeptidases that cause the degradation of extracellular matrix and connective tissue proteins [11]. MMPs are the major players in degrading the skeletal components of EG [24]. Syndecan-1 and syndecan-2 are shed by MMP-7 [25], and IL-1β increases the shedding of syndecan-4 by MMP-9 in immortalized glomerular endothelial cells [26]. Oxidative stress can enhance the activity of histone deacetylase (HDAC), up-regulate the expression and activity of MMPs, and down-regulate tissue inhibitor of matrix metalloproteinases (TIMPs), thus eventually lead to the loss of EG [27]. Furthermore, it was also found that syndecan-1 and SOD3 shedding could be reversed through inhibition of MMPs and HDAC [27].

The HDAC family is divided into four different subgroups. Subgroups I, II and IV are Zn^2+^-dependent enzymes, and subgroup III, Sirtuins (SIRTs), are nicotinamide adenine dinucleotide-dependent enzymes that are not affected by classical HDAC inhibitors [28]. The Sirtuins consist of seven proteins (SIRT1-SIRT7), all of which are involved in key processes related to health and longevity such as chromatin regulation, DNA repair and cell metabolism [29-31], among them, SIRT1 pathway plays a critical role in aging [29-31]. NF-κB is one of the target proteins of SIRT1 deacetylation [32]. Normally, NF-κB forms a dimer structure of p50-p65 (NF-κB1/RelA). In an inactive state, NF-κB binds to the specific inhibitor IκB protein in the cytoplasm and interferes with its nuclear translocation [33]. SIRT1 binds to the p65 protein subunit of NF-κB and deacetylates Lys310 in p65, resulting in the loss of NF-κB transcriptional activity [30]. Increased SIRT1 activity inhibits NF-κB-dependent inflammatory responses [34]. Syndecan-4 is the target gene of NF-κB. In SIRT1endo-/- mice, when nuclear translocation of NF-κB is increased, transcription of syndecan-4 is also elevated [30]. However, it was found that the expression of syndecan-4 isolated from renal microvascular endothelial cells of SIRT1endo-/- mice decreased and its extracellular domain increased concurrently, which may be related to the disruption of the integrity of EG transmembrane scaffold [35]. ADAM17 is known to shed the extracellular domain of Syndecan-4 [36]. Lack of SIRT1 induces an increase in ADAM17 activity, resulting in greater shedding of syndecan-4 [35]. Moreover, ADAM15 cleaves CD44 to promote increased vascular permeability [37]. HPSE is the only known enzyme that degrades HS [38] HA can be degraded by HAase or non-enzyme. The non-enzymatic degradation of HA is mainly caused by reactive oxygen species (ROS) [39].

## EG in Aging and Age-related Diseases

**Figure 1.**
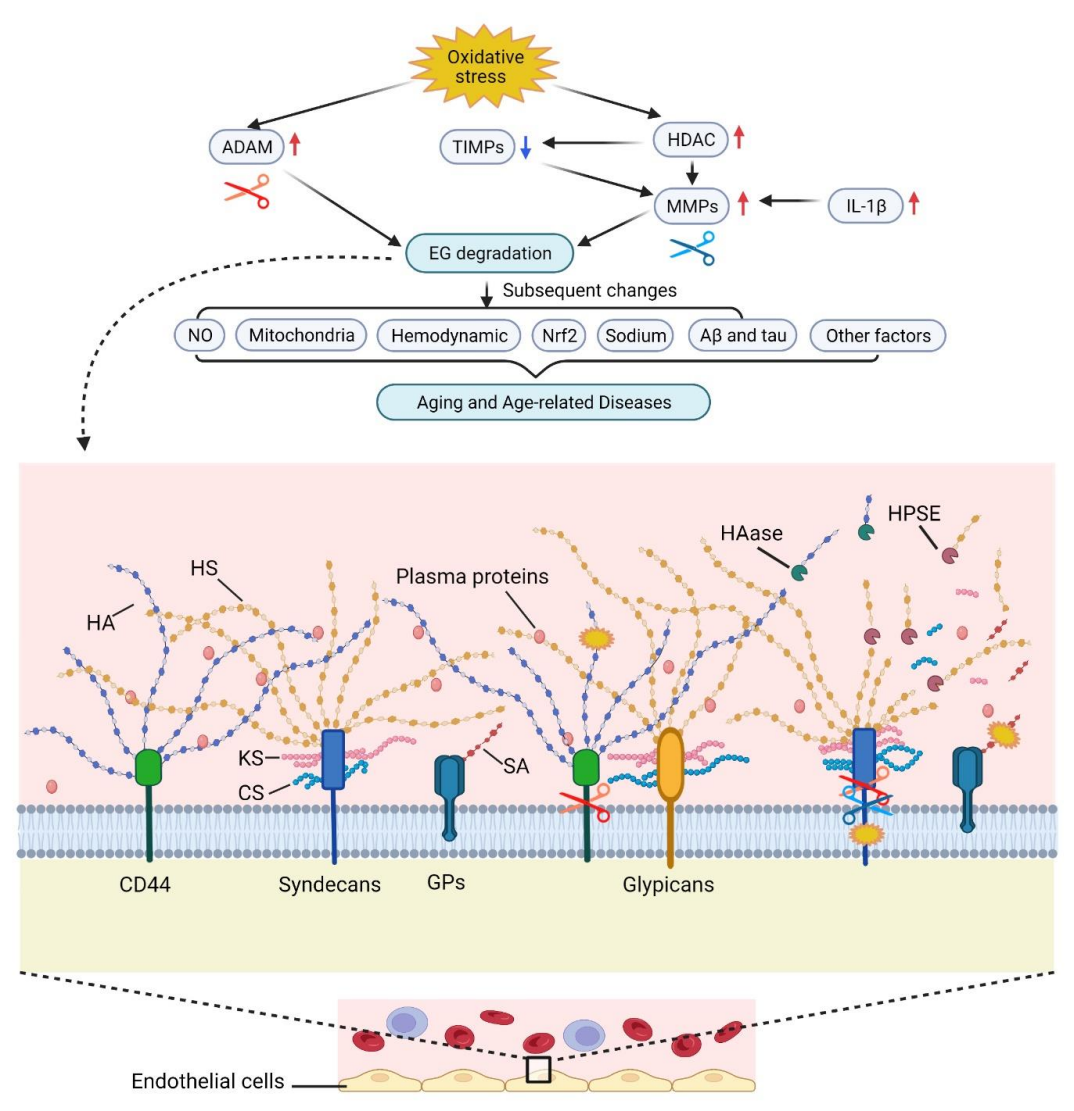
Endothelial Glycocalyx in Aging and Age-related Diseases. GPs, glycoproteins; HS, heparan sulfate; CS, chondroitin sulfate; KS, keratan sulfate; HA, hyaluronic acid; SA, sialic acid; HPSE, heparinase; HAase, hyaluronidase; EG, endothelial glycocalyx; MMPs, matrix metalloproteinases; ADAM, A disintegrin and metalloproteinase; HDAC, histone deacetylase; TIMPs, tissue inhibitor of matrix metalloproteinases; NO, nitric oxide; Nrf2, nuclear factor erythroid 2-related factor 2; Aβ, amyloid-beta. Created with BioRender.com.

Aging is associated with increased risk of a wide range of diseases and such diseases are labeled as age-related diseases. By definition, aging is a complex process characterized by progressive loss of physiological integrity, and increased susceptibility to age-related diseases and death [40]. Aging is also driven by the consecutive impairment of the microcirculation [41]. Age-related diseases can be defined as the continuous deterioration of tissues and organs over time, ultimately resulting in tissue and organ failure [42]. Twelve hallmarks of aging are proposed recently according to the following three criteria: (a) the time-dependent alterations manifestation during aging; (b) the possibility to accelerate aging by experimental aggravation; and (c) the opportunity to decelerate, halt, or reverse aging by therapeutic interventions [43]. Based on these criteria and the characteristics of EG, EG is a potential new marker of aging and age-related diseases (Fig. 1).

### Aging

Oxidative stress is widely recognized as an crucial factor associated with aging and age-related diseases [44]. EG components exposed to oxidative stress result in constant modifications in their structure, these changes in EG can be observed in aging. The expression of glypican-1, one of the main receptors that induce the regular release of NO [20], is inhibited in the aging process, in turn, vasoregulation can be damaged by reducing NO signaling [45]. Age-related deterioration of EG has been found in aged mice and humans. Studies showed that capillary density and EG thickness decreases in older adults, and the thickness of EG in sublingual microcirculation can decrease by 33% [46, 47]. In animal studies by observing the ultrastructure of rat tissue, it was found that EG thickness was decreased in aged rats [48]. It was also shown that the thickness of EG in the mesentery and skeletal muscle microvessels in aged mice is 51%-54% lower than that in young mice, which could be caused by age-related alterations in non-sulfated GAGs namely HA synthesis [47]. The same phenomenon was also found in aging mice, that degradation of HA increased in microvasculature of the cerebral cortex [49]. Similarly, SA is degraded from erythrocyte membranes at a higher rate in older adults compared to young adults, which may be associated with the increase in oxidative stress during aging [50]. In vitro study also has shown that aged cells have increased EG shedding and decreased EG layers, and under biomimetic shock conditions, EG shedding is further exacerbated in aged as compared to young endothelial cells [51]. Human brain is one of the two most abundant tissue sources of KS, and KS has neuroregulatory properties [52, 53]. The CS/KS PG aggrecan has anti-oxidant properties and play essential role in synaptic plasticity [52]. P-glycoprotein (P-gp) is expressed exclusively by endothelial cells in the brain as part of the blood-brain barrier (BBB). There is evidence that P-gp expression declines with age in the human BBB [54]. This is consistent with the decreasing trend of P-gp expression with age found in Fischer 344/Brown-Norway (F344/BN) aging model rats [55]. Overall, research on the direct role of EG in aging is still very limited.

### Cardiovascular diseases

The main features of the pathogenesis of cardiovascular diseases are oxidative stress, endothelial activation, and endothelial dysfunction [56]. Age advancement is the independent and essential prognosticator of endothelial dysfunction and vascular dysfunction [57, 58]. Oxidative stress, which has been described an imbalance favoring the generation of ROS and/or reducing antioxidant defense systems [59], is a major cause of endothelial dysfunction [56]. Oxidative stress is normally related to EG degradation, proteolysis and increased permeability in the cardiovascular system [60]. Studies have found the phenomenon of decreased EG in age-related vascular diseases [2, 3]. EG in healthy individuals contains around 1.5 liters of plasma, which can replenish the blood volume if needed [61], making EG a very powerful fluid reservoir. When EG thickness declines during the aging process, the capacity of the cardiovascular reserve and the circulatory system regulation also decreases. Deteriorated EG can affect the efficiency of blood flow distribution, and this can lead to the derangement of microvascular perfusion [62, 63]. Once the microcirculation is impaired, cells would not be able to obtain adequate nutrients and oxygen, and the physiological functions of cells and organs will deteriorate [41], which may ultimately result in tissue and organ failure. Therefore, EG deterioration could be one of the triggers of age-related vascular diseases. There is also evidence that supports a possible hypothesis that age-related EG worsens and causes microvascular dysfunction, which in turn leads to macrovascular dysfunction and eventually cardiovascular disease (CVD) [4]. The deterioration of EG may precede traditional measurements of age-related impairment of vascular function such as large artery stiffness [4]. This could be the pathological mechanism of EG in the development of age-related CVD. Indeed, a 6-year follow-up study have revealed that the damage of EG predicted future cardiovascular events [64]. Similarly, other study supports that EG could be a key therapeutic target of age-related cardiovascular diseases, and the mechanisms may be related to the decreased activation of nuclear factor erythroid 2-related factor 2 (Nrf2) caused by SA reduction, leading to the reduction of antioxidants [65]. It is well known that Nrf2 is a positive regulator of antioxidant pathways [65]. Moreover, animal experiments have also shown that decreased SIRT-1 expression in blood vessels of aged mice is associated with age-related atherosclerosis of the great arteries [66], and age-related aortic stiffness was attenuated in mice with lifelong SIRT-1 overexpression [67]. In view of the above evidence, it is necessary to conduct more studies to further examine the role of EG in age-related vascular diseases.

### Hypertension

Hypertension is a major risk factor for CVD, strokes, and has emerged as a pathogenic factor of Alzheimer’s disease (AD) [68, 69]. Excessive sodium intake has been shown to cause increase of blood pressure (BP) and been linked with hypertension and CVD [70, 71].The BP response to sodium is a predictor of severity of organ damage caused by hypertension [72] and sodium-dependent hypertension is related to EG degradation [73]. Importantly, GAGs are highly sulfated components with net negative charges, and have sodium-binding properties. Experiments in both mice and rats have identified that GAGs in the skin interstitium are responsible for sodium storage [74, 75], hence EG may have a significant role in sodium homeostasis. When EG integrity is lost, a sodium load would lead to water retention and increased BP [73]. NO is the major factor in maintaining vascular homeostasis and reduced bioavailability of NO marks the beginning of endothelial dysfunction [56]. In vitro studies have confirmed that on account of EG absence, plasma sodium concentration increases, which enhances endothelial stiffness and decreases the release of NO, eventually leading to elevated BP [76]. Study also showed in rat model of pulmonary arterial hypertension, heparin supplementation improved the integrity of EG and slowed down the development of hypertension [77]. This is also illustrated by the inverse correlation between EG volume and BP in patients with type II diabetes [78]. Besides, impaired EG and reduced thickness are associated with increased arterial stiffness in hypertensive patients [79, 80]. It was also found that hypertensive individuals have decreased erythrocyte membranes SA compared to normotensive individuals [50], and this supports that hemodynamic properties of the blood may be affected by decrease in SA content [81].

### Strokes

Strokes can be caused by imbalance in the regulation of oxidative stress, over production of ROS and mitochondrial dysfunction [44]. There are two types of strokes, the ischemic stroke and the hemorrhagic stroke. Ischemic stroke is caused by occlusion of cerebral arteries and accounts for approximately 87% of all strokes [82]. After stroke, astrocytes and microglia are rapidly activated, producing huge amounts of ROS, and causing BBB damage [83]. Impairment of the BBB integrity occurs early in the pathogenies of strokes. EG function as an essential component of the BBB [84], that in concert with the endothelial cell layer, abluminal basement membrane, and astrocyte endplates, plays an important role in the maintenance of BBB integrity [85]. Intact EG is critical for the modulation of BBB permeability [86]. Like studies in aging brains, technically it is challenging to study EG in the brains of strokes. In vivo measurement of EG in brain is almost impossible, hence sublingual microvasculature measurement of EG dimensions is used as an alternative method. Lacunar stroke patients with white matter lesions have an increased perfused boundary region (PBR) compared with healthy controls, which indicate EG impairment [87]. However, this phenomenon does not exist in lacunar stroke patients without white matter lesions, which could be due to the improvement of endothelial function by statins and antihypertensive [87, 88]. The shed EG components may serve as potential markers of EG impairment [89, 90]. The degree of EG damage can be assessed by measuring the levels of circulating EG components in plasma, such as syndecan-1, HS, CS [91]. And it has been shown that plasma level of syndecan-1 increased in acute ischemic stroke and syndecan-1 level predicted the prognosis in stroke patients [92]. The plasma level of SA is also an indicator of the degree of EG breakdown. The combination of elevated SA plasma levels and reduced SA erythrocyte concentrations is one of the markers of ischemic stroke [93]. Mitochondrial dysregulation is common in neurological diseases such as strokes [94, 95] and AD [96]. HA is hydrophilic [97], it binds to the osteopontin receptor CD44. HA-aggrecan interactions in brain protect mitochondria from oxidative damage [52]. Intriguingly, the synthesis of HA increases in the injured areas of the stroke brain [98, 99], and the reason may be related to CD44 affecting BBB function by regulating BBB permeability in response to fluid shear stress [100]. With current evidence from both animal and human studies, it appears that EG has great potential as a therapeutic target for BBB recovery following strokes.

### Neurodegenerative diseases

BBB breakdown in aging can lead to brain accumulation of serum proteins and several vasculotoxic and/or neurotoxic macromolecules, which contribute to the development of neuronal degenerative changes [44]. AD is the most common neurodegenerative diseases worldwide, characterized by the accumulation of amyloid-beta (Aβ) peptide and hyperphosphorylated tau [101, 102]. Oxidative stress is involved in the development of AD by facilitating Aβ deposition and the hyperphosphorylation of tau [103]. A human study found that EG was reduced in AD brains, leading to non-productive neutrophil adhesion to the vasculature and subsequent vascular oxidative stress [104]. HA level was elevated within damaged brain tissues in neurodegenerative diseases [105] and the increase of HA in AD brain was associated with Aβ and hyper-phosphorylated tau [106]. Interestingly, one study found that HA in cerebrospinal fluid (CSF) increased in vascular dementia patients as compared with controls while there was no difference between AD patients and controls [107]. There is a positive correlation between levels of HA in CSF and the CSF/ serum albumin ratio, an indicator of BBB integrity, in patients with vascular dementia and AD [107]. Furthermore, GAGs and PGs play pivotal roles in neuronal cell development and function [108, 109]. HSPGs are formed by the binding of HS to PGs [110]. HSPGS expression is altered in aging [45]. HSPGs may promote Aβ and tau fibrilization in AD brain [111] and it was observed the levels of some HSPGs were associated with lesions in AD [112]. This may be related to the fact that HSPGs play a role in synaptic stabilization [113, 114]. HS is relevant to almost every step of Aβ pathogenesis in AD [115]. It is possible that blocking the binding of Aβ to HS or desulfation of syndecan-2 can reduce the formation of amyloid plaques, and hence represent a potential therapeutic strategy for AD [116]. Given very limited and indefinitive findings on the role of EG in AD pathology, more research, especially human studies should be conducted in the future to shed light on the uncertainties.

## Regeneration of EG

EG plays an important role in maintaining physiological stability, therefore, regeneration of EG may be one potential solution to healthy aging. EG can be repaired by exogenous supplementation of EG components. Dietary supplementation with EG precursors has been shown to restore EG in patients with type II diabetes [78]. Previous study has also shown that dietary supplementation of EG precursor GP for 10 weeks in aged mice restored EG barrier function [117]. Endothelial vesicles contain abundant deposits of EG [118, 119], and it is hypothesized that the externalization of these substances can bring the components of EG back to the endothelial surface [120]. Cutler et al. have shown that metabolites of blueberries could restore GAGs, suggesting that blueberries could regenerate EG [121]. Therefore, older adults can increase blueberry intake in their diet to enhance their health and the effects could partially be attributed to regenerated EG.

Heparin and heparinoids may have a role in EG regeneration. Heparin is a sulfated glycine aminoglycan that can directly bind to endothelium to form EG [122], probably because it increases HS production [123]. Sulodexide (SDX) is a glycosaminoglycan mixture consisting of 80% HS and 20% DS [12] that provides precursors of HS and a material source for the restoration of EG [120]. Study has shown that SDX can reconstruct EG in a rat model with carotid artery balloon injury [124]. As a supplement of GAGs, SDX could enhance EG and activate the nuclear factor Nrf2 signal and provide protection against ischemia-reperfusion injury [125].

In addition, in vitro studies have shown that metformin could down-regulate the expression of ICAM and E-selectin and repair EG [126]. Plasma resuscitation was shown to enhance lung syndecan-1mRNA expression in a rat model of hemorrhagic shock, which is an early sign of EG recovery [127]. On the other hand, sphingosine-1-phosphate (S1P), a sphingolipid in the plasma mainly derived from red blood cells, can protect EG and maintain normal vascular permeability [128]. Clinical studies have also shown that albumin can bind to EG to stabilize its structure, reducing the degradation of EG by the associated enzymes [10]. Albumin acts on EG primarily through modulating S1P [129].

Physical training has also been shown to have a protective and pro-repair effect on EG, and this may contribute to its anti-aging effects. Short-term respiratory muscle training improves respiratory and functional capacity as well as reducing plasma syndecan-1 concentrations in hemodialysis patients [130]. High-intensity interval training increases EG thickness [131] while moderate-intensity endurance training protects EG by a mechanism that may be related to reduced oxidative stress and enhanced antioxidant defense [132].

In addition to nutritional, pharmacological, albumin, S1P-based and physical training interventions, there is also evidence that liposomal nanocarriers of preassembled EG were able to restore endothelial mechanotransduction [133]. It is possible that combining different approaches may achieve synergy in delaying aging and age-related diseases and future research should test different combinations.

## Conclusion

To date, there have been countless explorative efforts in finding solutions that can extend the human lifespan. With the economic developments and technological advances in medicine, people are not only concerned about extending lifespan but also paying more attention to improving the quality of their late-life. The concept of healthy aging has emerged in response to this changing era. The World Health Organization (WHO) first introduced the concept of healthy aging in 1990 and defined it in 2015 as a process of developing and maintaining bodily functions to enable older adults achieve well-being [134]. Diseases that come with aging affect every family. Although the question of whether the maximum human lifespan can be achieved remains open, the prevention, delay and, in some cases, reversal of the pathology of aging may be achieved through the study of physiopathology of aging, thus enabling older adults to live not only longer but also healthier lives [135]. One of the important pathophysiological processes of aging is the aging of blood vessels and the accumulation of vascular dysfunction [136]. EG is the main functional barrier to the vascular endothelium and plays an important role in maintaining selective vascular permeability [137]. Decreased microvascular density and loss of EG have been found during the aging process [51]. It has been shown in previous experiments that EG deficiency is closely linked to age-related diseases, thus protecting and repairing the EG is essential for healthy aging. Given the important role of EG in vascular homeostasis and the protection of various organs, as well as its regenerative properties [138], it is potentially an important therapeutic target for aging and age-related diseases. As previously mentioned, several drugs, foods and other measures have been shown to protect and restore EG. Although there is still a lack of definitive evidence, findings from early studies could guide future research on EG treatment. If more effective interventions can be developed to replenish EG in a timely manner, it may help to prevent age-related diseases and preserve physiological functions during human aging. Although many studies have greatly expanded our understanding of the role of EG in promoting healthy aging, much work remains to be done before we can clinically apply EG treatments to improve the health of older adults.
